# Sex Hormones in Allergic Conjunctivitis: Altered Levels of Circulating Androgens and Estrogens in Children and Adolescents with Vernal Keratoconjunctivitis

**DOI:** 10.1155/2015/945317

**Published:** 2015-01-28

**Authors:** Marta Sacchetti, Alessandro Lambiase, Costanzo Moretti, Flavio Mantelli, Stefano Bonini

**Affiliations:** ^1^Cornea and Ocular Surface Disease Unit, IRCCS Ospedale San Raffaele, Via Olgettina 60, 20132 Milan, Italy; ^2^Department of Organi di Senso, Sapienza University, Viale del Policlinico 155, 00161 Rome, Italy; ^3^Unit of Endocrinology, Fatebenefratelli Hospital, Isola Tiberina, University of Rome Tor Vergata, 00187 Rome, Italy; ^4^IRCCS GB Bietti Eye Foundation, Via Livenza 3, 00198 Rome, Italy; ^5^Department of Ophthalmology, Università Campus Bio-Medico, Via Álvaro del Portillo 21, 00128 Rome, Italy

## Abstract

*Purpose.* Vernal keratoconjunctivitis (VKC) is a chronic allergic disease mainly affecting boys in prepubertal age and usually recovering after puberty. To evaluate a possible role of sex hormones in VKC, serum levels of sex hormones in children and adolescents with VKC were assessed. *Methods.* 12 prepubertal and 7 early pubertal boys with active VKC and 6 male patients with VKC in remission phase at late pubertal age and 48 healthy age and sex-matched subjects were included. Serum concentration of estrone, 17 beta-estradiol, dehydroepiandrosterone-sulfate, total testosterone and free testosterone, dihydrotestosterone (DHT), cortisol, delta-4-androstenedione, follicle-stimulating hormone, luteinizing hormone, and sex-hormones binding globuline (SHBG) were evaluated. *Results.* Serum levels of Estrone were significantly increased in all groups of patients with VKC when compared to healthy controls (*P* < 0.001). Prepubertal and early pubertal VKC showed a significant decrease in DHT (*P* = 0.007 and *P* = 0.028, resp.) and SHBG (*P* = 0.01 and *P* = 0.002, resp.) when compared to controls and serum levels of SHBG were increased in late pubertal VKC in remission phase (*P* = 0.007). *Conclusions and Relevance.* VKC patients have different circulating sex hormone levels in different phases of the disease and when compared to nonallergic subjects. These findings suggest a role played by sex hormones in the pathogenesis and/or activity of VKC.

## 1. Introduction 

Vernal keratoconjunctivitis (VKC) is an ocular allergic disease mainly affecting the paediatric population, as it usually recovers spontaneously after puberty [1]. Similarly to other atopic conditions, VKC has a different prevalence between males and females. In particular, boys are two to four times more likely to develop VKC [1–4]. We previously described a significant increase of conjunctival estrogen and progesterone receptors in children with VKC as compared to healthy subjects. These findings, together with the higher disease prevalence in boys, suggest an influence of sex hormones on VKC development and/or activity [5].

In line with this, increasing evidence demonstrates a close relationship between sex hormones and ocular surface conditions such as dry eye, autoimmune diseases, contact lens intolerance, corneal angiogenesis, and wound healing [6].

Changes in sex hormone metabolism and balance may be a predisposing factor to immune-mediated diseases, including allergy [7, 8]. It is generally accepted that estrogens exert immunoenhancing activities, at least on the humoral immune response, while androgens exert immunosuppressive effects on both humoral and cellular immune responses and seem to represent natural anti-inflammatory hormones [8]. Particularly, androgens such as dehydroepiandrosterone (DHEA), dehydroepiandrosterone-sulfate (DHEA-S), and testosterone may influence the balance between type-1 and type-2 helper T cells (Th1/Th2), immunoglobulin (Ig) E synthesis, and eosinophil proliferation, thus modulating the development of allergic reactions [9–11]. On the other hand, estrogens may stimulate the allergic response by inducing synthesis of antibodies and by stimulating mast cell degranulation through estrogen receptor-alpha [8, 12]. Changes in circulating sex hormone levels and sex hormone-binding globulin (SHBG) have been described in patients with atopic dermatitis, asthma, hereditary angioneurotic oedema, and chronic urticaria [10, 13, 14]. However, circulating levels of sex hormones in patients with VKC have not yet been evaluated.

In the present study, serum concentration of estrone (E1), 17 beta-estradiol (E2), DHEA-S, total testosterone (TT), free testosterone (FT), dihydrotestosterone (DHT), cortisol, delta-4-androstenedione (D4A), follicle-stimulating hormone (FSH), luteinizing hormone (LH), and SHBG were evaluated in male patients with VKC in active- and remission-phase and were compared to healthy age and sex-matched subjects.

## 2. Methods

Nineteen consecutive male patients with active VKC (Bonini grade ≥ 1) were included in this study [15]. The patients were divided in 12 prepubertal boys (age range 6 to 9 years, LH < 1 IU/L, and TT < 30 ng/100 mL) and 7 early pubertal boys (*N* = 7, age range 10 to 12 years, LH < 3 IU/L, and TT < 200 ng/100 mL). Six additional late pubertal (14 to 17 years of age, LH 1 to 10 IU/L and TT > 200 ng/100 mL) patients with VKC in remission phase (Bonini grade < 1) were included (Table 1).

Twenty healthy nonallergic prepubertal males along with 16 early pubertal and 12 late pubertal males were used as controls. Written informed consent for participation was obtained from their parents according to the local requirements. The study was performed in accordance with the tenants of the Declaration of Helsinki following approval of the local IRB.

Girls with VKC were not included in this study to avoid the confounding factor of sex hormones blood levels differences due to gender. Patients with ocular and/or systemic associated diseases were excluded with the exception of associated atopic diseases. None of the patients or healthy subjects were taking or previously took systemic steroids, phenytoin, or other medications known to interfere with sex hormone metabolism.

Vernal Keratoconjunctivitis diagnosis was based on a history of ocular surface inflammation characterised by itching, photophobia, tearing and mucous discharge, the presence of a giant papillary reaction on the upper tarsal conjunctiva, and/or at the limbus associated with the presence of eosinophils in conjunctival scrapings.

The following clinical and demographic data of VKC patients were collected: age, duration of disease, history of associated atopic diseases, and positive reaction to skin PRICK tests.

Disease activity was graded according to the Bonini VKC severity score, as follows: grade 0 (quiescent) = absence of ocular symptoms; grade 1 (mild) = presence of ocular symptoms but not photophobia; grade 2 (moderate) = presence of symptoms and photophobia; grade 3 (moderate) = presence of ocular symptoms and mild-to-moderate superficial punctuate keratitis (SPK); grade 4 (very severe) = presence of diffuse SPK and/or corneal ulcer [15]. Patients with past history of VKC, absent or mild-intermittent ocular symptoms, and the presence of conjunctival papillary reaction associated with subconjunctival fibrosis were considered in the remission phase of the disease [16].

Blood samples were obtained in the morning from all subjects. Sex hormone serum concentrations were performed following the standard hospital procedures by the Clinical Laboratory of the University of Tor Vergata. In particular, estrone (E1), 17-beta estradiol (E2), DHEA-S, TT, FT, DHT, FSH, and LH, and delta-4-androstenedione were determined by radio-immunoassay (RIA) and serum concentration of SHBG was performed by immunoradiometric assay (IRMA). The total serum IgE concentration (PRIST) was also assessed by radio-immunoassay (RIA).

The serum concentrations of sex hormones in patients with VKC were compared to those of age-matched controls by independent samples *t*-test. Clinical grade, demographic characteristics, and IgE concentration were related with sex hormone levels by Spearman rho test and independent samples *t*-test (SPSS for Windows, V.15, SPSS, Inc., Chicago, IL, USA). *P* values below 0.05 were considered statistically significant.

## 3. Results 

Twenty-five consecutive male VKC patients were included in this study. Clinical characteristics of patients with VKC included in the study are listed in Table 1.

Patients with VKC in active phase (*N* = 19) showed decreased serum levels of DHT, SHBG, and FSH (*P* < 0.001, *P* < 0.001, and *P* < 0.001, resp.) and increased levels of estrone and FT (*P* < 0.001, *P* = 0.007, resp.) when compared to age-matched healthy subjects (Table 2). These changes were confirmed when patients with VKC were divided in prepubertal (*N* = 12) and early pubertal (*N* = 7) groups. Particularly, serum DHT and SHBG concentration were decreased and serum estrone was increased in both the prepubertal (*P* = 0.007, *P* = 0.01, and *P* < 0.001, resp.) and early pubertal VKC groups (*P* = 0.028, *P* = 0.002, and *P* = 0.001, resp.) when compared to age-matched healthy subjects. Early pubertal VKC patients also showed increased serum concentrations of FT (*P* = 0.002) and early pubertal patients showed decreased FSH serum levels (*P* = 0.002) when compared to healthy subjects.

Patient with VKC in the remission phase showed increased serum concentration of SHBG and estrone (*P* = 0.007 and *P* = 0.007, resp.) and no significant difference in DHT concentration when compared to healthy subjects. In addition, a significant decrease of FT and DHEA-S (*P* = 0.033, *P* = 0.012, resp.) levels were observed when compared to healthy controls (Table 3).

Patients with positive reaction to skin prick test showed a significantly higher estrone serum concentration when compared to patients with no skin reaction, regardless of age and stage of VKC activity. In particular, estrone serum concentration in patients with active VKC was 40 ± 14 pg/mL in patients with positive skin test, as compared to 23 ± 12 pg/mL in VKC with negative skin test (*P* = 0.044). Patients with VKC in remission phase also showed higher estrone levels in the group with positive skin prick test as compared to negative skin prick test (43 ± 3 pg/mL versus 31 ± 6 pg/mL, *P* = 0.049). No significant correlations were observed between serum IgE levels, clinical grade of VKC, and sex hormones levels.

## 4. Discussion 

In this study we demonstrated that boys with active VKC have different circulating sex hormone levels as compared to healthy subjects. VKC is a chronic allergic disease, and as in other atopic conditions such as atopic eczema and asthma, boys are more frequently affected compared to girls, with this ratio becoming almost equal after puberty due to a spontaneous remission in the late puberty. It has been hypothesized that the recovery of VKC at puberty may be the result of a protective function of androgens [1, 2, 17, 18]. Both androgens and estrogens exert a role in immune diseases. Although the precise mechanisms by which these steroid hormones affect the immune system are not completely elucidated, their effects may be responsible for the gender differences in the onset and course of immune diseases, including allergic conditions [8, 17, 19, 20].

Our data show that children with VKC have differences in circulating sex hormone levels when compared to age and sex-matched healthy subjects that are potentially responsible for disease activity. In line with our hypothesis of a role played by sex hormones in the pathogenesis and remission of VKC, in this study we also observed that late pubertal (adolescent) VKC patients with disease in remission phase did not show the same differences in sex hormones' pattern when compared to healthy controls. Particularly, pre- and early pubertal boys with active VKC showed a decrease in DHT and SHBG associated with an increase in Estrone serum concentrations, while late pubertal boys with VKC in remission showed a normalization of DHT and an increase in estrone and SHBG serum levels.

All patients with active VKC—regardless of age—showed a consistent significant reduction of DHT, the more active androgen. DHT is synthesized from testosterone in peripheral tissues by the 5*α*-reductase enzyme [21]. DHT is able to depress the systemic immune response, and it has been demonstrated that DHT plays a major role in suppressing leukotriene production, a key molecule in the allergic reaction [22]. It has been hypothesized that DHT constitutes a molecular basis for gender difference in inflammatory diseases such as asthma. The decrease of serum DHT in patients with active VKC as compared to healthy subjects may be responsible for a lack in the androgen protective function, which, in turn, could play a role in the age- and gender-related differences observed in VKC development. However, DHT levels do not correlate with VKC severity grade, suggesting that this steroid hormone may predispose to developing VKC rather than influencing directly the severity of the inflammatory reaction. The decrease of DHT in VKC patients may result from changes in circulating sex hormones carriers, such as SHBG, or it may reflect an alteration of sex hormone pathways. SHBG is a glycoprotein known to bind to circulating testosterone, DHT, and estradiol, which physiologically decreases at puberty in association with the increase in circulating sex hormone levels [23, 24]. In our VKC patients, the lower levels of DHT do not appear to be related to changes in serum SHBG concentration. In fact, children with VKC showed a decrease in SHBG levels when compared to healthy subjects. These changes in SHBG may explain the increase of FT levels observed in active VKC. In line with this hypothesis, late pubertal VKC in remission phase showed a significant increase of SHBG concentration associated with lower FT and DHEA-S. The low level of DHEA-S in late pubertal patients with VKC may be also caused by a decrease of adrenal hormones production. Some evidence suggests that inflammatory conditions may be associated with reduced adrenal hormones production and their blood levels when compared to healthy controls [8, 25, 26]. However, DHEA-S levels were not changed in patients with VKC in the active phase when compared to healthy controls.

In line with the overall hypothesis of an involvement of sex hormones in VKC, circulating estrone levels were increased in all VKC groups when compared to healthy subjects. This observation is in line with the well-known role of estrogens in allergic diseases, including eosinophil chemotaxis and immune reactions' triggering [27]. Our observation that VKC patients with a positive prick skin test reaction had the highest circulating levels of estrone strongly supports the role of this hormone in allergic reactions.

Our results on the changes in circulating sex hormone levels in VKC patients suggest that sex hormone receptors or pathways at conjunctival inflammatory sites may represent an additional potential therapeutic target for ocular allergic diseases.

## Figures and Tables

**Table 1:** Clinical characteristics of patients included in this study.

	Total	Prepuberty	Early puberty	Late puberty
Patients *N*	25	12	7	6
Atopic associated diseases (*N*)				
Rhinitis	2	0	2	0
Asthma	5	2	1	2
Atopic dermatitis	2	2	0	0
Atopic dermatitis and asthma	4	1	1	2
None	12	7	3	2
Skin prick test (*N*)				
Positive	14	7	3	4
Negative	11	5	4	2
Total IgE (U/mL)	215 ± 257	323 ± 347	261 ± 189	111 ± 76
Duration of VKC (years)				
Mean ± SD	5.6 ± 3.4	3 ± 1.4	6.1 ± 2.2	10 ± 2.6
VKC type (*N*)				
Tarsal	22	11	7	4
Limbal	3	1	0	2
Mixed	0	0	0	0
VKC course				
Seasonal	14	6	5	3
Perennial	11	6	2	3
VKC grade				
0—quiescent	6	0	0	6
1—mild	2	2	0	0
2—moderate	9	6	3	0
3—severe	4	1	3	0
4—very severe	4	3	1	0

SD: standard deviation; VKC: vernal keratoconjunctivitis.

**Table 2:** Sex hormones serum levels in active VKC and healthy subjects.

	Total		Prepubertal		Early pubertal	
	VKC	Healthy	VKC	Healthy	VKC	Healthy
*N*	19	36	12	20	7	16
Age						
Mean ± SD	8.7 ± 2	8.5 ± 2.9	7.3 ± 0.9	7 ± 1.3	11 ± 0.7	11 ± 1.1
Range	6–12	6–13	6–9	6–9	9–12	9–13
LH (mUI/mL)						
Mean ± SD	0.5 ± 0.3	0.6 ± 0.5	0.4 ± 0.2	0.4 ± 0.2	0.9 ± 0.5	0.8 ± 0.5
Range	0.2–1.2	0.1–2	0.2–0.7	0.1–0.8	0.5–1.2	0.1–2
FSH (mUI/mL)						
Mean ± SD	0.7 ± 0.5^*^	**1.4 ± 0.7**	0.6 ± 0.4	1 ± 0.6	0.8 ± 0.6^*^	**1.8 ± 0.8**
Range	**0.3–1.7**	**0.2–4**	0.3–1.2	0.2–3	**0.3–1.7**	**0.6–4**
Total testosterone (ng/100 mL)						
Mean ± SD	28.7 ± 47	18 ± 12	13 ± 9.1	9.8 ± 4.2	36 ± 24	26 ± 12
Range	1–73	4–48	1–27	4–17	7–73	6–48
Free testosterone (ng/100 mL)						
Mean ± SD	2.7 ± 4.7^*^	**1 ± 1**	2 ± 2.8^*^	**0.4 ± 0.1**	4.1 ± 7.4	1.6 ± 1
Range	**0.5–19.2**	**0.2–4.9**	**0.5–8.6**	**0.2–0.5**	0.5–19.2	0.2–4.9
DHT (pg/mL)						
Mean ± SD	17 ± 11.7	41.7 ± 36.3	15 ± 8.7^*^	**21 ± 4**	21 ± 19^*^	**64 ± 43**
Range	3–38	15–140	**3–25**	**15–27**	**1.3–38**	**20–140**
Estradiol (pg/mL)						
Mean ± SD	14.7 ± 6.8	17.7 ± 5.5	14.9 ± 7.2	17.4 ± 4.9	14.2 ± 6.7	17.9 ± 6
Range	5–29	10–34	5–29	10–27	10–24	10–34
Estrone (pg/mL)						
Mean ± SD	33.8 ± 18.8^*^	**12.8 ± 2.4**	33.7 ± 3.5^*^	**12 ± 1.7**	34.5 ± 3.5^*^	**16.5 ± 1.9**
Range	**10–64**	**10–18**	**10–64**	**10–15**	**32–37**	**14–18**
DHEAS (ng/mL)						
Mean ± SD	855 ± 1008	612 ± 221	707 ± 685	488 ± 186	1111 ± 1440	731 ± 186
Range	87–2640	132–1200	87–2190	132–861	273–2640	358–1200
D4A (ng/mL)						
Mean ± SD	0.7 ± 0.7	0.7 ± 0.3	0.7 ± 0.8	0.5 ± 0.2	0.9 ± 0.4	0.9 ± 0.4
Range	0.1–2.5	0.3–2.2	0.1–2.5	0.3–0.9	0.6–1.2	0.6–2.2
Cortisol (ng/mL)						
Mean ± SD	122 ± 73	102 ± 49	138 ± 80	107 ± 55	79 ± 28	96 ± 41
Range	31–238	35–205	31–238	35–205	51–107	45–197
SHBG (nmol/L)						
Mean ± SD	**65.9 ± 41**	120 ± 19^*^	76.6 ± 42.6^*^	**110.5 ± 28**	41.7 ± 25^*^	**134 ± 49**
Range	**21–164**	**49–208**	**40–164**	**84–208**	**21–78**	**49–206**

VKC: vernal keratoconjunctivitis; ^*^ *P* < 0.05; *N*: number; SD: standard deviation; LH: luteinizing hormone; FSH: follicle-stimulating hormone; DHT: dihydrotestosterone; DHEAS: dehydroepiandrosterone-sulfate; D4A: delta-4-androstenedione; SHBG: sex hormones binding globulin.

**Table 3:** Sex hormones serum levels in patients with VKC in remission phase compared to healthy subjects.

	Late puberty	
	VKC	Healthy
*N*	6	12
Age		
Mean ± SD	14–17	14–17
Range	15.3 ± 1	15.7 ± 1
LH (mUI/mL)		
Mean ± SD	2.7 ± 0.6	2.6 ± 1.4
Range	2.1–3.5	1.1–5.4
FSH (mUI/mL)		
Mean ± SD	5.8 ± 3.8	5.2 ± 1.5
Range	1.4–8.2	2.9–9.8
Total testosterone (ng/100 mL)		
Mean ± SD	369 ± 142	369 ± 142
Range	202–593	254–789
Free testosterone (ng/100 mL)		
Mean ± SD	17.1 ± 42^*^	**24.8 ± 6.6**
Range	**202–593**	**254–789**
DHT (pg/mL)		
Mean ± SD	362 ± 37	445 ± 89
Range	320–386	289–593
Estradiol (pg/mL)		
Mean ± SD	21 ± 9	27 ± 7
Range	15–37	19–41
Estrone (pg/mL)		
Mean ± SD	36.5 ± 7^*^	**25.5 ± 2.7**
Range	**27–43**	**22–30**
DHEAS (ng/mL)		
Mean ± SD	1131 ± 700^*^	**1783 ± 398**
Range	**645–2370**	**1047–2648**
D4A (ng/mL)		
Mean ± SD	1.4 ± 0.5	1.6 ± 0.3
Range	1–2.2	1.1–2.1
Cortisol (ng/mL)		
Mean ± SD	130 ± 63	119 ± 37
Range	56–209	76–203
SHBG (nmol/L)		
Mean ± SD	73 ± 44^*^	**30 ± 9**
Range	**36–123**	**13–39**

VKC: vernal keratoconjunctivitis; ^*^ *P* < 0.05; *N*: number; SD: standard deviation; LH: luteinizing hormone; FSH: follicle-stimulating hormone; DHT: dihydrotestosterone; DHEAS: dehydroepiandrosterone-sulfate; D4A: delta-4-androstenedione; SHBG: sex hormones binding globulin.
